# Syntheses, crystal structures and Hirshfeld surface analysis of 4-(4-nitro­phen­yl)piperazin-1-ium tri­fluoro­acetate and 4-(4-nitro­phen­yl)piperazin-1-ium tri­chloro­acetate

**DOI:** 10.1107/S2056989022011501

**Published:** 2023-01-01

**Authors:** Holehundi J. Shankara Prasad, Devaraju, Subbaiah M. Murthy, Hanna Kaspiaruk, Hemmige S. Yathirajan, Sabine Foro, Lilianna Chęcińska

**Affiliations:** a Department of Chemistry, Yuvaraja’s College, University of Mysore, Mysore 570 005, India; b Department of Microbiology, Yuvaraja’s College, University of Mysore, Mysore 570 005, India; c Faculty of Chemistry, University of Lodz, Pomorska 163/165, 90-236 Łódź, Poland; d Department of Studies in Chemistry, University of Mysore, Manasagangotri, Mysuru 570 006, India; e Institute of Materials Science, Darmstadt University of Technology, Alarich-Weiss-Strasse 2, D-64287 Darmstadt, Germany; Katholieke Universiteit Leuven, Belgium

**Keywords:** crystal structure, piperazine, hydrogen bonding, chain of rings, supra­molecular assembly

## Abstract

The synthesis and crystal structures of the mol­ecular salts of 4-(4-nitro­phen­yl)piperazine with tri­fluoro­acetate, namely, 4-(4-nitro­phen­yl)piperazin-1-ium tri­fluoro­acetate, C_10_H_14_N_3_O_2_^+^·C_2_F_3_O_2_^−^ (I), and with tri­chloro­acetate, namely, 4-(4-nitro­phen­yl)piperazin-1-ium tri­chloro­acetate, C_10_H_14_N_3_O_2_^+^·C_2_Cl_3_O_2_^−^, (II), are reported and compared. A partial positional disorder of the anions was found. In both structures, the piperazine rings adopt a chair conformation, whereas the positions of the nitro­phenyl group on the piperazine ring differ from bis­ectional in (I) to equatorial in (II). In both structures, the supra­molecular assemblies are mono-periodic on the basis of the chain-of-rings motifs supported by aromatic π–π inter­actions. Hirshfeld surface analysis was used to explore the inter­molecular close contacts in both crystals. The most dominant contacts of the Hirshfeld surface of the cation–anion pairs of the asymmetric units are O⋯H/H⋯O, and those with a contribution of halogen atoms: F⋯H/H⋯F in (I) and Cl⋯H/H⋯Cl in (II), respectively.

## Abstract 2

The supra­molecular assemblies of the two title structures are one-dimensional: the chain-of-rings motifs are supported by aromatic π–π inter­actions.

## 1. Chemical context

Piperazines and their derivatives have attracted growing attention for years (Berkheij *et al.*, 2005 ▸; Elliott, 2011 ▸; Asif, 2015 ▸; Brito *et al.*, 2019 ▸), mainly because of their multivalent biological profiles in a number of different therapeutic areas (Upadhayaya *et al.*, 2004 ▸; Chaudhary *et al.*, 2006 ▸; Kharb *et al.*, 2012 ▸). The pharmacological significance of piperazines is also manifested in the application of its framework in the assemblies of inclusion, hybrid and other functional materials (Brockunier *et al.*, 2004 ▸; Bogatcheva *et al.*, 2006 ▸; Jin *et al.*, 2020 ▸; Gharbi *et al.*, 2022 ▸). Among them, a potential application for 4-nitro­phenyl­piperazine (NPP) can be indicated (König *et al.*, 1997 ▸; Lu, 2007 ▸; Wang *et al.*, 2014 ▸). We have recently reported the crystal structures of eight salts of 4-nitro­phenyl­piperazine (Mahesha *et al.*, 2022 ▸; Shankara Prasad *et al.*, 2022 ▸). In view of the importance of piperazines in general and the use of 4-nitro­phenyl­piperazine in particular, the present article reports the synthesis, crystal structure and Hirshfeld surface analysis of two salts of 4-nitro­phenyl­piperazine with organic acids, namely, 4-(4-nitro­phen­yl)piperazin-1-ium tri­fluoroacetate, C_12_H_14_F_3_N_3_O_4_, (I) and 4-(4-nitro­pheny)piperazin-1-ium tri­chloro­acetate, C_12_H_14_Cl_3_N_3_O_4_, (II). 
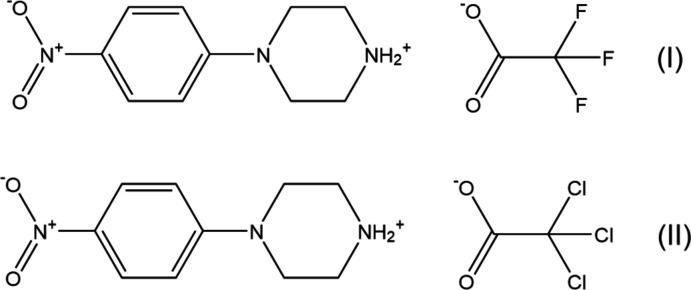


## 2. Structural commentary

**Figure 1:**
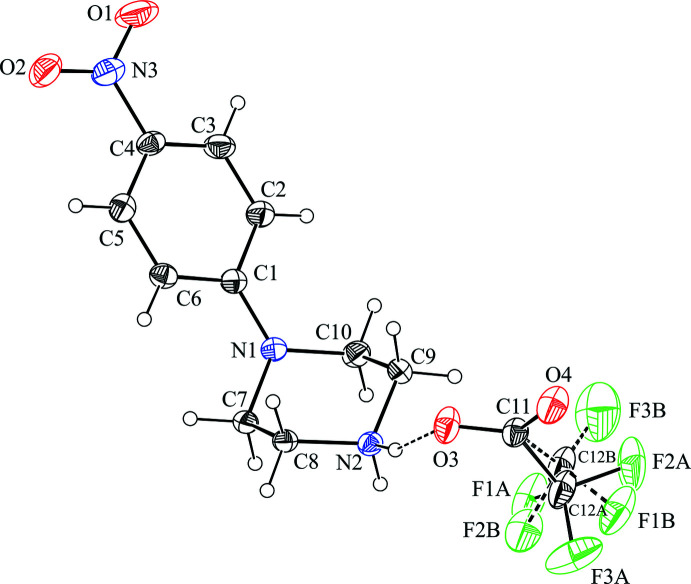
Independent components of compound (I) showing the atom-labelling scheme and the hydrogen bond (drawn as dashed line) within the selected asymmetric unit. The major disorder component is drawn using unbroken lines (*A*) and the minor disorder component is drawn using dashed lines (*B*). Displacement ellipsoids are drawn at the 30% probability level.

**Figure 3:**
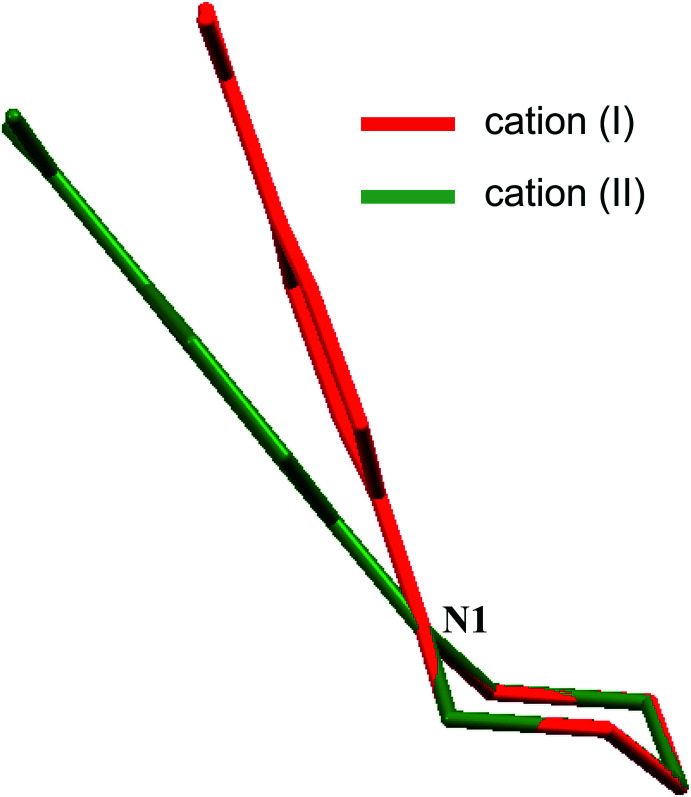
Superposition of the 4-(4-nitro­phen­yl)piperazin-1-ium cations in (I) (red) and (II) (green).

The title compounds are shown in Figs. 1 ▸ and 2 ▸. The piperazine rings adopt a chair conformation with puckering parameters (Cremer & Pople, 1975 ▸) in (I) of *Q* = 0.576 (2) Å, θ = 177.8 (2)°, φ = 182 (4)°, and in (II) of *Q* = 0.571 (2) Å, θ = 177.1 (2)°, φ = 189 (4)°, respectively. The position of the nitro­phenyl group on the piperazine ring differs in the two structures, from bis­ectional in (I) to occupying an equatorial site in (II) (Fig. 3 ▸). The angle between the N1—C1 bond and the normal to the Cremer & Pople mean plane is 39.57 (11)° in (I) and 60.87 (14)° in (II) (Spek, 2020 ▸; see *Database survey* section for further comparisons). In addition, the delocalization effect within the benzene ring is slightly disturbed due to the presence of the electron-donating piperazinyl [–C_4_H_8_N_2_; for the structurally similar piperidino substituent the Hammett σ_p_ constant is −0.12 (Perrin *et al.*, 1981 ▸)] and the electron-withdrawing nitro [–NO_2_, σ_p_ = 0.78 (Hansch *et al.*, 1991 ▸)] groups located in the *para*- position: the lengthening of the C1—C2 and C1—C6 bonds is accompanied by the shortening of the remaining C—C bonds within the ring and C—N distances to the substituents.

In the anions, the C—O bond lengths in the carboxyl­ate group are more similar in compound (II) than in compound (I), although in both cases these distances are shorter than the mean value for its type (Allen *et al.*, 1987 ▸). The geometries of the COO^−^ groups can be affected by the positional disorder of the CF_3_ group in (I) and the chlorine atoms in (II). In (I), the CF_3_ group is found to be disordered over two orientations, with a refined occupancy ratio of 0.779 (4):0.221 (4), while in (II), the disordered chlorine atoms in the CCl_3_ group show an almost equivalent contribution of components *A* and *B* [0.494 (15) and 0.506 (15)] (Figs. 1 ▸ and 2 ▸).

## 3. Supra­molecular features

**Figure 4:**
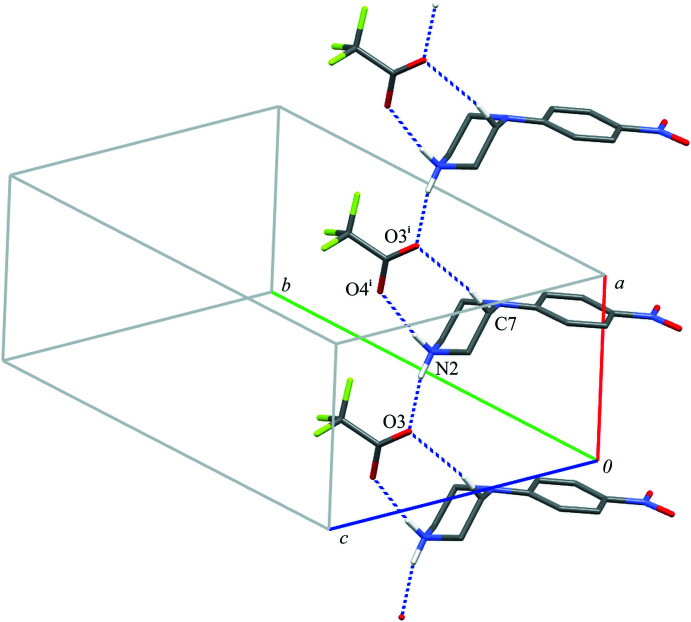
Part of the crystal structure of compound (I) showing the formation of a chain of rings parallel to the [100] direction. Hydrogen bonds are drawn as dashed lines, and for the sake of clarity, the H atoms bonded to C atoms have been omitted. Symmetry code: (i) *x* + 1, *y*, *z*.

In (I), the 4-(4-nitro­phen­yl)piperazin-1-ium cation inter­acts with two tri­fluoro­acetate anions, which are related by translation, by two N—H⋯O hydrogen bonds: N2—H21⋯O3 and N2—H21⋯O4(*x* + 1, *y*, *z*). Additionally, if one considers the C7—H7*A*⋯O3(*x* + 1, *y*, *z*) inter­action the hydrogen-bonded motif can be described as a *C*(6)*C*(6)[ 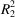 (8)] chain of rings (Etter, 1990 ▸; Etter *et al.*, 1990 ▸; Bernstein *et al.*, 1995 ▸) running parallel to the [100] direction (Fig. 4 ▸, Table 1 ▸).

**Figure 5:**
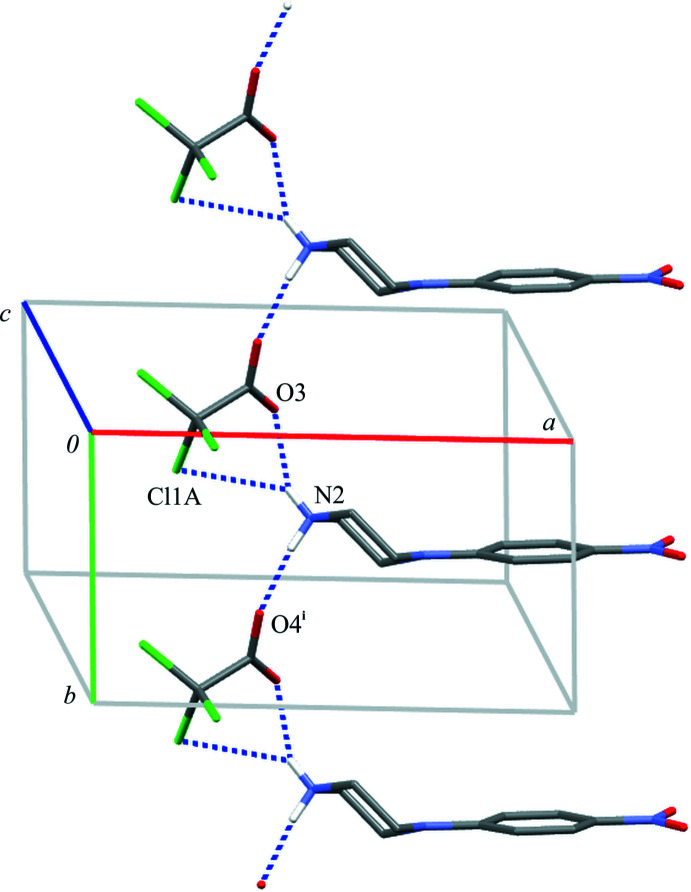
Part of the crystal structure of compound (II) showing the formation of a chain of rings parallel to the [010] direction. Hydrogen bonds are drawn as dashed lines, and for the sake of clarity, the H atoms bonded to C atoms have been omitted. Symmetry code: (i) *x*, *y* + 1, *z*.

In (II), the ionic components of the asymmetric unit are linked by two N2—H21⋯O3 and N2—H21⋯Cl1*A* hydrogen bonds, forming an 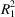 (5) ring motif. This ring system is further propagated along the [010] direction through the N2—H22⋯O3(*x*, *y* + 1, *z*) hydrogen bond; and a *C*(6)*C*(7)[ 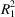 (5)] chain of rings is created (Fig. 5 ▸, Table 2 ▸).

**Figure 6:**
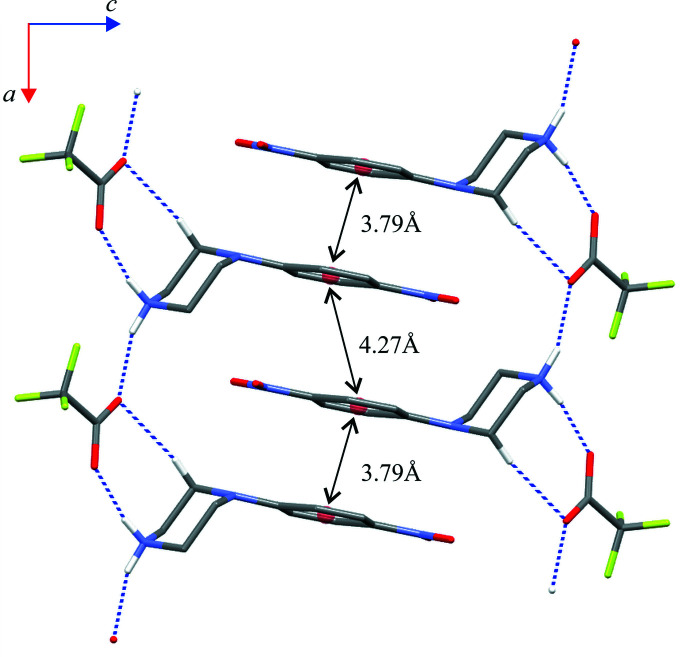
A part of the crystal structure of compound (I) showing the aromatic π–π inter­actions between adjacent chains of rings. Red balls represent the centroids of the phenyl rings (*Cg*1).

Close inspection of the crystal packings of both structures reveals the aromatic π–π inter­actions between adjacent chains of rings (Figs. 6 ▸ and 7 ▸). The centroid–centroid distances (*Cg*1⋯*Cg*1) between the phenyl rings are 3.788 (1) and 4.268 (1) Å in (I) and 3.800 (1) Å in (II); the perpendicular distances from the centroid to the plane of the opposite ring are 3.333 (1) and 3.253 (1) Å in (I) and 3.303 (1) Å in (II). Although in (I) the slippage distance (2.764 Å) between the centroids spaced by 4.27 Å is markedly far from a value of 1.8 Å (suggesting an overlap of rings), one can still consider mol­ecular stacks along the [100] direction to be comparable to those undoubtedly observed in structure (II) in the [010] direction.

**Figure 8:**
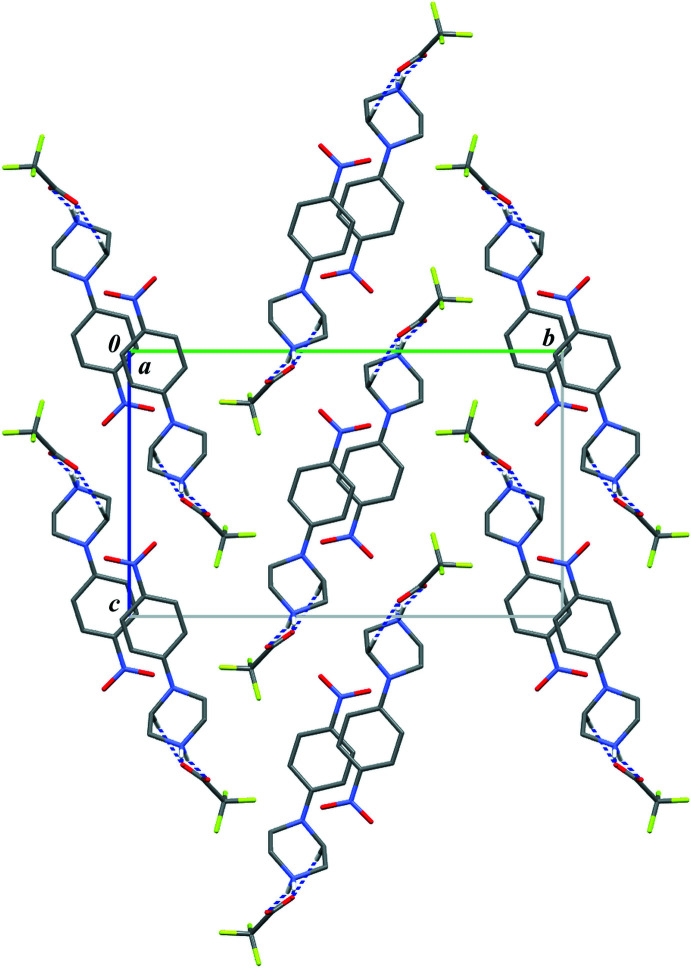
Crystal packing of (I) in a view along the crystallographic *a* axis (herring-bone type).

**Figure 9:**
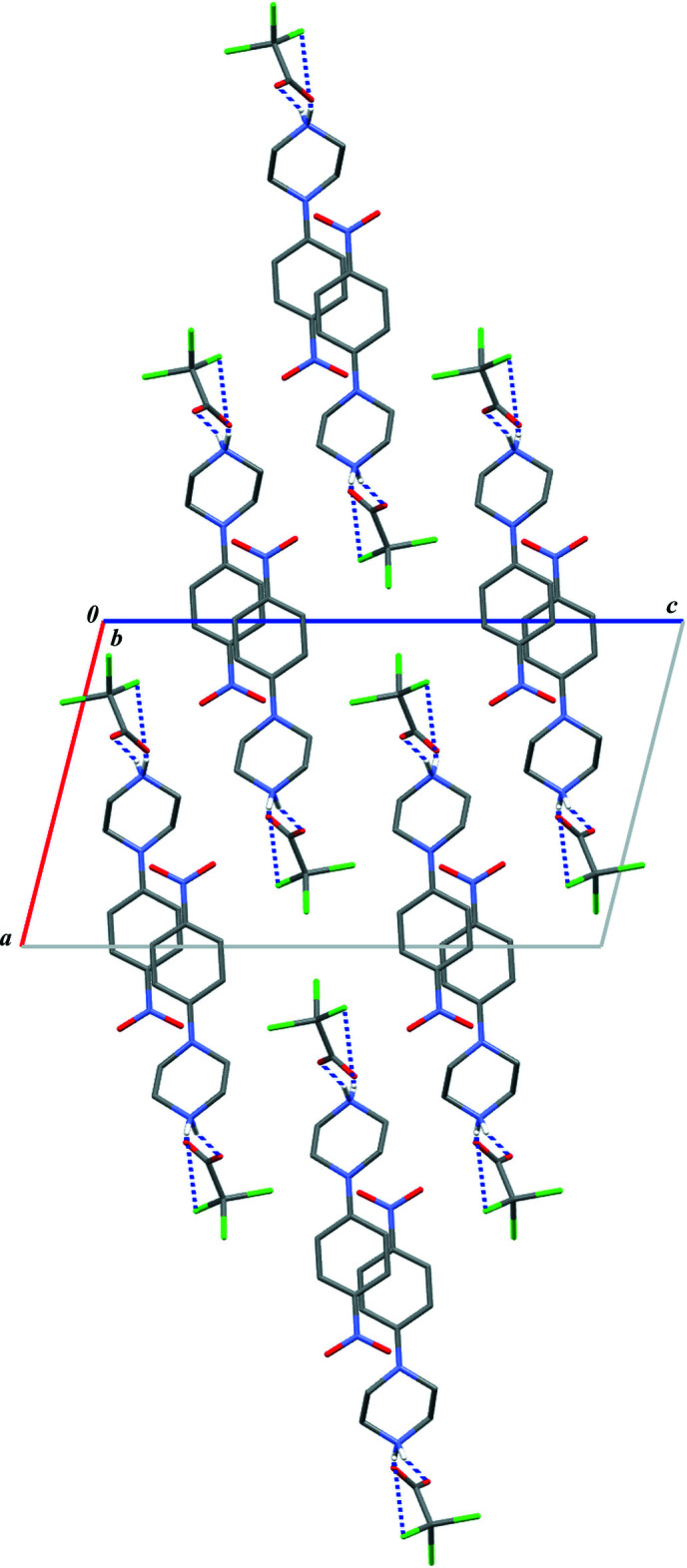
Crystal packing of (II) in a view along the crystallographic *b* axis (linear type).

Finally, both supra­molecular structures can be described as mono-periodic; no other specific close contacts or inter­actions can be found in addition to those mentioned above. Despite the similarities in the formation of 1D-chains of rings and their stacking assemblies, the packing of these motifs in the analysed crystals is fundamentally different. In (I), the packing fashion can be described as herringbone-type (Fig. 8 ▸), whereas in (II) a linear mode is seen (Fig. 9 ▸). It seems that the halogen atoms [F in (I) and Cl in (II)] in the anions influence the crystal-packing modes because of the difference in their van der Waals radii.

## 4. Hirshfeld surface analysis

**Figure 10:**
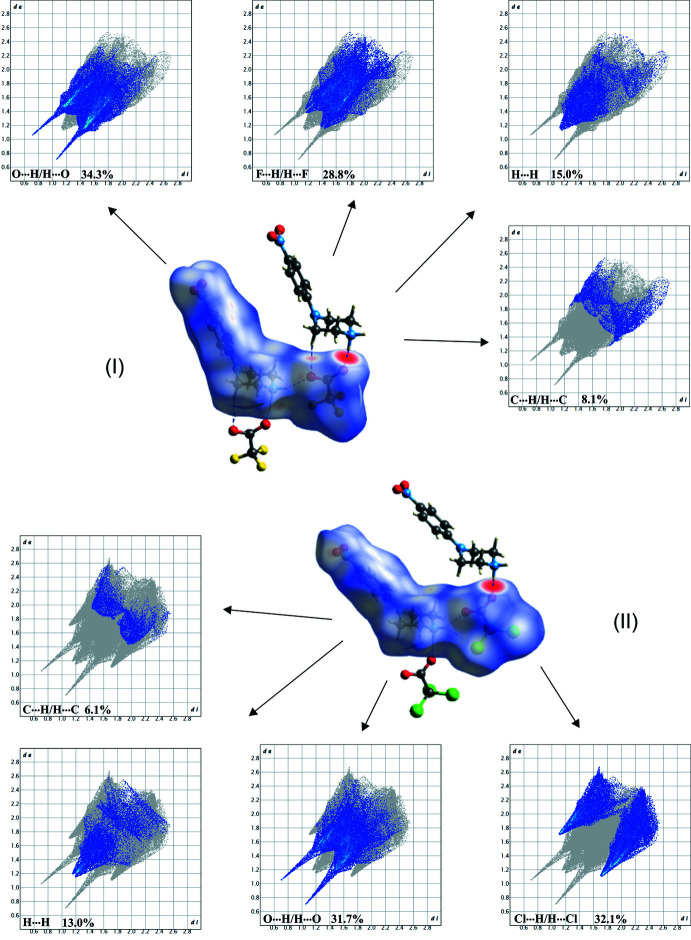
Views of the Hirshfeld surfaces of the ionic components of (I) (upper) and (II) (lower) mapped over *d*_norm_ showing inter­molecular hydrogen bonds as dashed lines. Hirshfeld surface analysis were carried out using *CrystalExplorer* (Spackman & Jayatilaka, 2009 ▸; Turner *et al.*, 2017 ▸).

The Hirshfeld surface analysis is a valuable tool for understanding crystal packing. It offers both identification and visualization of inter­molecular inter­actions, as well as reflecting the inter­play between atoms in the structure. The Hirshfeld surfaces of ionic pairs in the asymmetric units of (I) and (II), are shown in Fig. 10 ▸. In addition, in Fig. 10 ▸, the corresponding 2D fingerprint plots of the most dominant contacts are also presented and combined with the information about their percentage contributions to the Hirshfeld surface. For both structures, the most significant contacts percentages are attributed to O⋯H/H⋯O inter­actions, 34.3% in (I) and 31.7% in (II). The closest contacts of this type appear as two sharp symmetric spikes in the 2D maps, and the inter­molecular contacts as representatives are visualized between the Hirshfeld surface of the ionic components and neighbouring mol­ecules. Competing close contacts are those with halogen atom, Cl⋯H/H⋯Cl type in (I) (32.1%) and F⋯H/H⋯F in (II) (28.8%). The former contacts in the fingerprint plot of (II) can be seen as wings, whereas the latter contacts dominate in the structure of (I) are spread over the central part of plot; their distances are essentially comparable or longer than the sum of the van der Waals radii of the atoms involved. The much lower contributions of the H⋯H contacts are consistent with the moderate number of H atoms per two mol­ecules in the asymmetric units. The contributions of the remaining contact types constitute about 20%, among which 6–8% of the Hirshfeld surface area of (I) and (II) is covered by C⋯H/H⋯C contacts.

## 5. Database survey

**Figure 11:**
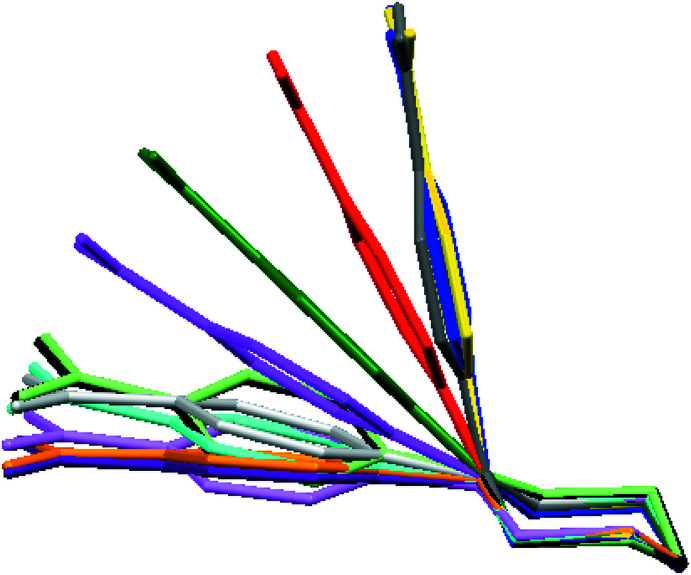
An overlay of thirteen 4-(4-nitro­phen­yl)piperazin-1-ium cations, showing the best fit for the piperazine ring: the colour code is red = (I), green = (II[Chem scheme1]), orange = BEFGIG, blue = BEFGOM, black = NEBVOJ, light green = NEBVUP, purple = NEBWAW, cyan = NEBWEA, light grey = NEBWIE (mol­ecule 1), grey = NEBWIE (mol­ecule 2), violet = NEBWOK (mol­ecule 1), magenta = NEBWOK (mol­ecule 2) and yellow = LIJNAU.

A search of the Cambridge Structural Database (CSD version 5.43, September 2022; Groom *et al.* 2016 ▸) for 4-nitro­phenyl­piperazines in organic compounds revealed 45 structures, most of which contain a substituent at the N2 atom. Only a few compounds are directly comparable to title compounds (I) and (II): eight structures of 4-nitro­phenyl­piperazin-1-ium salts with different benzoate anions (NEBVOJ; NEBVUP; NEBWAW; NEBWEA; NEBWIE; NEBWOK; Mahesha *et al.*, 2022 ▸; BEFGIG; BEFGOM, Shankara Prasad *et al.*, 2022 ▸) and one with chloride (LIJNAU; Lu, 2007 ▸). In addition, two neutral NPP mol­ecules have been reported in an inclusion material (König *et al.*, 1997 ▸) or co-crystal (Wang *et al.*, 2014 ▸). We have compared the mol­ecular conformation of thirteen independent 4-(4-nitro­phen­yl)piperazin-1-ium cations: nine published structures (2 with *Z*′ > 1) and the two reported in this article. As shown in Fig. 11 ▸, the mol­ecular structures of the NPP cations differ from each other with respect to the position of the nitro­phenyl group on the piperazine ring: the equatorial site is preferred (9/13), whereas the axial position (3/13) is rare, and bis­ectional is uncommon (1/13). All compared piperazine rings adopt a chair conformation.

## 6. Synthesis and crystallization

A solution of commercially available (from Sigma-Aldrich) 4-nitro­phenyl­piperazine (100 mg, 0.483 mol) in methanol (10 ml) was mixed with equimolar solutions of the appropriate acids in methanol (10 ml) *viz*., tri­fluoro­acetic acid (55 mg, 0.483 mol) for (I) and tri­chloro­acetic acid (79 mg, 0.483 mol) for (II). The corresponding solutions were stirred for 30 minutes at 323 K and allowed to stand at room temperature. X-ray quality crystals were formed on slow evaporation for a week for both of the compounds, where ethanol ethyl acetate (1:1) was used for crystallization. The corresponding melting points were 425–427 K (I) and 388–390 K (II).

## 7. Refinement

Crystal data, data collection and structure refinement details for both compounds are summarized in Table 3 ▸. In both structures, an extinction parameter was refined.

The CF_3_ group of (I) was found to be disordered over two orientations, with a refined occupancy ratio of 0.779 (4):0.221 (4). The disorder was restrained using SIMU, ISOR and DELU commands in *SHELXL* for the six resulting fluorine atoms. Anisotropic displacement parameters for pairs of the disordered carbon atom (C12*A* and C12*B*) were constrained to be the same. The three C—F bonds of the minor disorder component (*B*) and two C11—C12 bonds were restrained to be similar in length.

In (II), the refined occupancies of disordered chlorine atoms in the CCl_3_ group of 0.494 (15) and 0.506 (15), show the equivalent contribution of the components *A* and *B*. The ellipsoids of three chlorine atoms of the *B* disorder component were modelled using SIMU, ISOR and DELU commands in *SHELXL*. All six C—Cl distances were restrained to be similar in length.

In both structures, the H atoms bound to C atoms were positioned geometrically with C—H distances of 0.93 Å (aromatic) and 0.97 Å (CH_2_), and with *U*_iso_(H) = 1.2*U*_eq_(C). The positions of the NH_2_ hydrogen atoms were refined. N—H distances within the NH_2_ group were restrained to 0.87 (2) Å.

## Supplementary Material

Crystal structure: contains datablock(s) I, II, global. DOI: 10.1107/S2056989022011501/vm2275sup1.cif

Structure factors: contains datablock(s) I. DOI: 10.1107/S2056989022011501/vm2275Isup2.hkl

Structure factors: contains datablock(s) II. DOI: 10.1107/S2056989022011501/vm2275IIsup3.hkl

Supporting information file. DOI: 10.1107/S2056989022011501/vm2275Isup4.cml

Supporting information file. DOI: 10.1107/S2056989022011501/vm2275IIsup5.cml

CCDC references: 2223441, 2223442

Additional supporting information:  crystallographic information; 3D view; checkCIF report

## Figures and Tables

**Figure 2:**
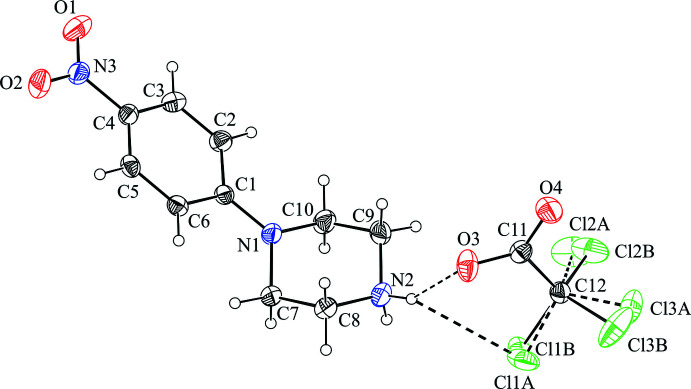
Independent components of compound (II) showing the atom-labelling scheme and the hydrogen bonds (drawn as dashed lines) within the selected asymmetric unit. The disorder components *A* and *B* of chlorine atoms have equal site-occupancies (1/2) within s.u. Displacement ellipsoids are drawn at the 30% probability level.

**Figure 7:**
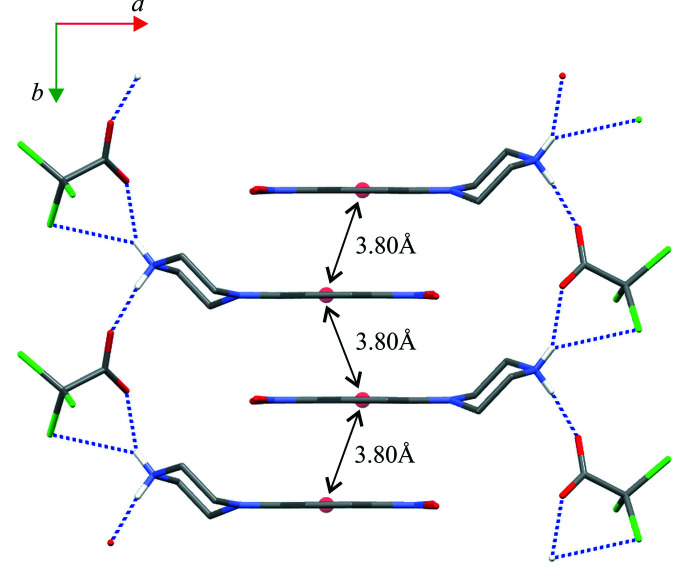
A part of the crystal structure of compound (II) showing the aromatic π–π inter­actions between adjacent chains of rings. Red balls represent the centroids of the phenyl rings (*Cg*1).

**Table 1:** Hydrogen-bond geometry (Å, °) for (I)

*D*—H⋯*A*	*D*—H	H⋯*A*	*D*⋯*A*	*D*—H⋯*A*
N2—H21⋯O3	0.90 (2)	1.98 (2)	2.844 (2)	161 (2)
N2—H22⋯O4^i^	0.87 (2)	1.93 (2)	2.786 (2)	165 (2)
C7—H7*A*⋯O3^i^	0.97	2.53	3.492 (2)	169

Symmetry code: (i) 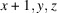 .

**Table 2:** Hydrogen-bond geometry (Å, °) for (II)

*D*—H⋯*A*	*D*—H	H⋯*A*	*D*⋯*A*	*D*—H⋯*A*
N2—H21⋯O3	0.87 (2)	2.01 (2)	2.795 (2)	151 (2)
N2—H21⋯Cl1*A*	0.87 (2)	2.82 (2)	3.510 (5)	138 (2)
N2—H22⋯O4^i^	0.87 (2)	1.89 (2)	2.738 (2)	167 (2)

Symmetry code: (i) 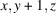 .

**Table 3:** Experimental details

	(I)	(II)
Crystal data		
Chemical formula	C_10_H_14_N_3_O_2_^+^·C_2_F_3_O_2_^−^	C_10_H_14_N_3_O_2_^+^·C_2_Cl_3_O_2_^−^
*M*_r_	321.26	370.61
Crystal system, space group	Monoclinic, *P*2_1_/*c*	Monoclinic, *P*2_1_/*c*
Temperature (K)	293	293
*a*, *b*, *c* (Å)	6.6889 (4), 18.376 (1), 11.2600 (7)	11.7825 (5), 6.6142 (3), 20.3271 (9)
β (°)	91.131 (6)	104.173 (4)
*V* (Å^3^)	1383.76 (14)	1535.91 (12)
*Z*	4	4
Radiation type	Mo *K*α	Mo *K*α
μ (mm^−1^)	0.14	0.62
Crystal size (mm)	0.50 × 0.44 × 0.44	0.48 × 0.44 × 0.40

Data collection		
Diffractometer	Oxford Diffraction Xcalibur with Sapphire CCD	Oxford Diffraction Xcalibu with Sapphire CCD
Absorption correction	Multi-scan (*CrysAlis RED*; Oxford Diffraction (2009 ▸)	Multi-scan (*CrysAlis RED*; Oxford Diffraction (2009 ▸)
*T*_min_, *T*_max_	0.784, 1.000	0.840, 1.000
No. of measured, independent and observed [*I* > 2σ(*I*)] reflections	4855, 2515, 1908	5078, 2802, 2068
*R*_int_	0.020	0.013

Refinement		
*R*[*F* ^2^ > 2σ(*F* ^2^)], *wR*(*F* ^2^), *S*	0.041, 0.106, 1.02	0.036, 0.104, 1.09
No. of reflections	2515	2802
No. of parameters	239	236
No. of restraints	84	35
H-atom treatment	H atoms treated by a mixture of independent and constrained refinement	H atoms treated by a mixture of independent and constrained refinement
Δρ_max_, Δρ_min_ (e Å^−3^)	0.28, −0.28	0.25, −0.28

Computer programs: *CrysAlis CCD* and *CrysAlis RED* (Oxford Diffraction, 2009 ▸), *SHELXT* (Sheldrick, 2015*a* ▸), *SHELXL2019/2* (Sheldrick, 2015*b* ▸), *Mercury* (Macrae *et al.*, 2020 ▸) and *publCIF* (Westrip, 2010 ▸).

## supplementary crystallographic information

### 4-(4-Nitrophenyl)piperazin-1-ium trifluoroacetate (I) Crystal data

**Table:**

C_10_H_14_N_3_O_2_^+^·C_2_F_3_O_2_^−^	*F*(000) = 664
*M**_r_* = 321.26	*D*_x_ = 1.542 Mg m^−^^3^
Monoclinic, *P*2_1_/*c*	Mo *K*α radiation, λ = 0.71073 Å
*a* = 6.6889 (4) Å	Cell parameters from 2766 reflections
*b* = 18.376 (1) Å	θ = 2.9–27.8°
*c* = 11.2600 (7) Å	µ = 0.14 mm^−^^1^
β = 91.131 (6)°	*T* = 293 K
*V* = 1383.76 (14) Å^3^	Prism, yellow
*Z* = 4	0.50 × 0.44 × 0.44 mm

### 4-(4-Nitrophenyl)piperazin-1-ium trifluoroacetate (I) Data collection

**Table:**

Oxford Diffraction Xcalibur with Sapphire CCD diffractometer	1908 reflections with *I* > 2σ(*I*)
Radiation source: Enhance (Mo) X-ray Source	*R*_int_ = 0.020
Rotation method data acquisition using ω scans.	θ_max_ = 25.3°, θ_min_ = 2.9°
Absorption correction: multi-scan (CrysAlis RED; Oxford Diffraction (2009)	*h* = −8→7
*T*_min_ = 0.784, *T*_max_ = 1.000	*k* = −17→22
4855 measured reflections	*l* = −11→13
2515 independent reflections	

### 4-(4-Nitrophenyl)piperazin-1-ium trifluoroacetate (I) Refinement

**Table:**

Refinement on *F*^2^	Hydrogen site location: mixed
Least-squares matrix: full	H atoms treated by a mixture of independent and constrained refinement
*R*[*F*^2^ > 2σ(*F*^2^)] = 0.041	*w* = 1/[σ^2^(*F*_o_^2^) + (0.0484*P*)^2^ + 0.5034*P*] where *P* = (*F*_o_^2^ + 2*F*_c_^2^)/3
*wR*(*F*^2^) = 0.106	(Δ/σ)_max_ < 0.001
*S* = 1.02	Δρ_max_ = 0.28 e Å^−^^3^
2515 reflections	Δρ_min_ = −0.28 e Å^−^^3^
239 parameters	Extinction correction: *SHELXL2019/2* (Sheldrick, 2015b), Fc^*^=kFc[1+0.001xFc^2^λ^3^/sin(2θ)]^-1/4^
84 restraints	Extinction coefficient: 0.053 (3)
Primary atom site location: structure-invariant direct methods	

### 4-(4-Nitrophenyl)piperazin-1-ium trifluoroacetate (I) Special details

**Table:**

Geometry. All esds (except the esd in the dihedral angle between two l.s. planes) are estimated using the full covariance matrix. The cell esds are taken into account individually in the estimation of esds in distances, angles and torsion angles; correlations between esds in cell parameters are only used when they are defined by crystal symmetry. An approximate (isotropic) treatment of cell esds is used for estimating esds involving l.s. planes.

### 4-(4-Nitrophenyl)piperazin-1-ium trifluoroacetate (I) Fractional atomic coordinates and isotropic or equivalent isotropic displacement parameters (Å^2^)

**Table:**

	*x*	*y*	*z*	*U*_iso_*/*U*_eq_	Occ. (<1)
O1	0.6694 (3)	0.05792 (11)	−0.27331 (14)	0.0805 (6)	
O2	0.6790 (2)	−0.05336 (10)	−0.21928 (14)	0.0636 (5)	
N1	0.8545 (2)	0.10090 (8)	0.26900 (12)	0.0356 (4)	
N2	0.6570 (2)	0.12077 (8)	0.48752 (13)	0.0312 (4)	
N3	0.6925 (2)	0.01145 (11)	−0.19696 (15)	0.0476 (5)	
C1	0.8114 (2)	0.07873 (10)	0.15542 (15)	0.0312 (4)	
C2	0.8000 (3)	0.12928 (11)	0.06160 (16)	0.0419 (5)	
H2	0.818859	0.178501	0.077433	0.050*	
C3	0.7615 (3)	0.10711 (12)	−0.05228 (17)	0.0447 (5)	
H3	0.753987	0.141171	−0.113294	0.054*	
C4	0.7339 (3)	0.03448 (11)	−0.07686 (15)	0.0365 (4)	
C5	0.7466 (2)	−0.01673 (11)	0.01221 (16)	0.0377 (4)	
H5	0.729993	−0.065848	−0.005234	0.045*	
C6	0.7840 (3)	0.00528 (10)	0.12678 (16)	0.0355 (4)	
H6	0.791371	−0.029400	0.186860	0.043*	
C7	0.8875 (3)	0.05084 (10)	0.36718 (15)	0.0341 (4)	
H7A	1.001562	0.066979	0.414794	0.041*	
H7B	0.917467	0.002845	0.336392	0.041*	
C8	0.7060 (3)	0.04672 (9)	0.44371 (15)	0.0320 (4)	
H8A	0.593779	0.027324	0.397946	0.038*	
H8B	0.732201	0.014504	0.510461	0.038*	
C9	0.6318 (3)	0.17363 (10)	0.38863 (16)	0.0373 (4)	
H9A	0.610217	0.221963	0.420550	0.045*	
H9B	0.515721	0.160535	0.340249	0.045*	
C10	0.8162 (3)	0.17372 (10)	0.31302 (16)	0.0401 (5)	
H10A	0.796762	0.206785	0.246641	0.048*	
H10B	0.930352	0.190504	0.359862	0.048*	
C11	0.1577 (3)	0.17011 (10)	0.61221 (17)	0.0386 (5)	
C12A	0.2811 (6)	0.2193 (2)	0.6965 (3)	0.0574 (11)	0.779 (4)
C12B	0.295 (2)	0.2305 (8)	0.6565 (13)	0.0574 (11)	0.221 (4)
O3	0.2518 (2)	0.12351 (8)	0.55930 (14)	0.0522 (4)	
O4	−0.0240 (2)	0.18168 (9)	0.61548 (14)	0.0593 (5)	
F1A	0.4768 (4)	0.22180 (19)	0.6652 (3)	0.0873 (11)	0.779 (4)
F2A	0.2265 (4)	0.28753 (13)	0.6917 (4)	0.0974 (13)	0.779 (4)
F3A	0.2611 (5)	0.2012 (2)	0.8079 (2)	0.1261 (13)	0.779 (4)
F1B	0.2251 (14)	0.2549 (8)	0.7591 (12)	0.089 (3)	0.221 (4)
F2B	0.4517 (17)	0.1991 (8)	0.7164 (11)	0.090 (3)	0.221 (4)
F3B	0.2926 (17)	0.2875 (5)	0.5852 (13)	0.129 (3)	0.221 (4)
H21	0.541 (3)	0.1200 (11)	0.5272 (17)	0.047 (6)*	
H22	0.752 (2)	0.1340 (10)	0.5372 (15)	0.038 (5)*	

### 4-(4-Nitrophenyl)piperazin-1-ium trifluoroacetate (I) Atomic displacement parameters (Å^2^)

**Table:**

	*U*^11^	*U*^22^	*U*^33^	*U*^12^	*U*^13^	*U*^23^
O1	0.1184 (16)	0.0917 (14)	0.0310 (8)	−0.0055 (11)	−0.0098 (9)	0.0050 (9)
O2	0.0640 (10)	0.0726 (12)	0.0539 (10)	0.0033 (8)	−0.0089 (8)	−0.0253 (8)
N1	0.0441 (9)	0.0359 (8)	0.0268 (8)	0.0041 (7)	0.0012 (6)	0.0023 (7)
N2	0.0272 (8)	0.0362 (8)	0.0301 (8)	0.0006 (7)	−0.0020 (6)	−0.0032 (7)
N3	0.0359 (9)	0.0719 (13)	0.0349 (9)	0.0018 (9)	−0.0004 (7)	−0.0080 (9)
C1	0.0234 (8)	0.0406 (10)	0.0298 (9)	0.0020 (7)	0.0030 (7)	0.0014 (8)
C2	0.0518 (12)	0.0397 (11)	0.0341 (10)	0.0012 (9)	0.0002 (8)	0.0025 (9)
C3	0.0508 (12)	0.0527 (13)	0.0306 (10)	0.0033 (10)	−0.0012 (8)	0.0079 (9)
C4	0.0277 (9)	0.0528 (12)	0.0289 (9)	0.0020 (8)	−0.0003 (7)	−0.0037 (9)
C5	0.0269 (9)	0.0433 (11)	0.0429 (11)	−0.0001 (8)	0.0005 (8)	−0.0064 (9)
C6	0.0317 (9)	0.0404 (11)	0.0344 (10)	0.0005 (8)	0.0001 (7)	0.0038 (8)
C7	0.0342 (9)	0.0388 (10)	0.0291 (9)	0.0075 (8)	−0.0028 (7)	0.0002 (8)
C8	0.0358 (9)	0.0312 (9)	0.0288 (9)	0.0000 (8)	−0.0048 (7)	0.0006 (7)
C9	0.0438 (11)	0.0314 (10)	0.0363 (10)	0.0062 (8)	−0.0056 (8)	0.0005 (8)
C10	0.0523 (11)	0.0346 (10)	0.0335 (10)	−0.0058 (9)	−0.0005 (8)	0.0011 (8)
C11	0.0313 (10)	0.0398 (11)	0.0446 (11)	−0.0015 (8)	−0.0029 (8)	−0.0100 (9)
C12A	0.0369 (14)	0.068 (2)	0.067 (3)	0.0037 (13)	0.0025 (19)	−0.036 (2)
C12B	0.0369 (14)	0.068 (2)	0.067 (3)	0.0037 (13)	0.0025 (19)	−0.036 (2)
O3	0.0386 (8)	0.0484 (8)	0.0695 (10)	0.0011 (7)	−0.0020 (7)	−0.0248 (8)
O4	0.0308 (8)	0.0745 (11)	0.0724 (11)	−0.0014 (7)	−0.0055 (7)	−0.0315 (8)
F1A	0.0316 (11)	0.103 (2)	0.128 (3)	−0.0196 (12)	0.0112 (14)	−0.0703 (18)
F2A	0.0739 (15)	0.0495 (14)	0.168 (4)	0.0015 (12)	−0.012 (2)	−0.0513 (17)
F3A	0.146 (3)	0.168 (3)	0.0625 (16)	−0.035 (2)	−0.0317 (15)	−0.0260 (18)
F1B	0.055 (4)	0.112 (6)	0.100 (5)	−0.010 (5)	0.006 (4)	−0.068 (4)
F2B	0.036 (4)	0.120 (5)	0.111 (5)	0.015 (4)	−0.032 (4)	−0.057 (4)
F3B	0.126 (5)	0.083 (5)	0.178 (6)	−0.035 (4)	0.003 (5)	−0.029 (5)

### 4-(4-Nitrophenyl)piperazin-1-ium trifluoroacetate (I) Geometric parameters (Å, º)

**Table:**

O1—N3	1.219 (2)	C7—C8	1.505 (2)
O2—N3	1.220 (2)	C7—H7A	0.9700
N1—C1	1.368 (2)	C7—H7B	0.9700
N1—C10	1.452 (2)	C8—H8A	0.9700
N1—C7	1.452 (2)	C8—H8B	0.9700
N2—C9	1.485 (2)	C9—C10	1.512 (3)
N2—C8	1.486 (2)	C9—H9A	0.9700
N2—H21	0.901 (15)	C9—H9B	0.9700
N2—H22	0.873 (15)	C10—H10A	0.9700
N3—C4	1.439 (2)	C10—H10B	0.9700
C1—C6	1.399 (3)	C11—O3	1.224 (2)
C1—C2	1.408 (2)	C11—O4	1.235 (2)
C2—C3	1.365 (3)	C11—C12B	1.518 (15)
C2—H2	0.9300	C11—C12A	1.539 (4)
C3—C4	1.375 (3)	C12A—F2A	1.306 (5)
C3—H3	0.9300	C12A—F3A	1.307 (5)
C4—C5	1.377 (3)	C12A—F1A	1.364 (5)
C5—C6	1.370 (3)	C12B—F3B	1.319 (13)
C5—H5	0.9300	C12B—F1B	1.332 (14)
C6—H6	0.9300	C12B—F2B	1.365 (14)
			
C1—N1—C10	123.95 (15)	N2—C8—C7	109.25 (14)
C1—N1—C7	123.34 (15)	N2—C8—H8A	109.8
C10—N1—C7	110.45 (13)	C7—C8—H8A	109.8
C9—N2—C8	111.88 (13)	N2—C8—H8B	109.8
C9—N2—H21	107.2 (13)	C7—C8—H8B	109.8
C8—N2—H21	110.4 (13)	H8A—C8—H8B	108.3
C9—N2—H22	111.7 (13)	N2—C9—C10	109.95 (15)
C8—N2—H22	107.8 (12)	N2—C9—H9A	109.7
H21—N2—H22	107.9 (18)	C10—C9—H9A	109.7
O1—N3—O2	122.05 (18)	N2—C9—H9B	109.7
O1—N3—C4	118.42 (19)	C10—C9—H9B	109.7
O2—N3—C4	119.53 (19)	H9A—C9—H9B	108.2
N1—C1—C6	121.82 (16)	N1—C10—C9	110.05 (15)
N1—C1—C2	120.84 (17)	N1—C10—H10A	109.7
C6—C1—C2	117.31 (16)	C9—C10—H10A	109.7
C3—C2—C1	120.96 (18)	N1—C10—H10B	109.7
C3—C2—H2	119.5	C9—C10—H10B	109.7
C1—C2—H2	119.5	H10A—C10—H10B	108.2
C2—C3—C4	120.06 (18)	O3—C11—O4	130.58 (17)
C2—C3—H3	120.0	O3—C11—C12B	111.0 (6)
C4—C3—H3	120.0	O4—C11—C12B	116.9 (6)
C3—C4—C5	120.72 (17)	O3—C11—C12A	115.9 (2)
C3—C4—N3	119.83 (18)	O4—C11—C12A	113.3 (2)
C5—C4—N3	119.45 (18)	F2A—C12A—F3A	104.5 (4)
C6—C5—C4	119.45 (18)	F2A—C12A—F1A	103.1 (4)
C6—C5—H5	120.3	F3A—C12A—F1A	112.0 (4)
C4—C5—H5	120.3	F2A—C12A—C11	113.1 (3)
C5—C6—C1	121.49 (17)	F3A—C12A—C11	112.2 (3)
C5—C6—H6	119.3	F1A—C12A—C11	111.5 (3)
C1—C6—H6	119.3	F3B—C12B—F1B	105.1 (13)
N1—C7—C8	110.83 (14)	F3B—C12B—F2B	129.5 (15)
N1—C7—H7A	109.5	F1B—C12B—F2B	89.6 (11)
C8—C7—H7A	109.5	F3B—C12B—C11	112.5 (10)
N1—C7—H7B	109.5	F1B—C12B—C11	108.2 (11)
C8—C7—H7B	109.5	F2B—C12B—C11	107.8 (11)
H7A—C7—H7B	108.1		
			
C10—N1—C1—C6	−157.34 (17)	C10—N1—C7—C8	61.08 (19)
C7—N1—C1—C6	3.9 (3)	C9—N2—C8—C7	54.85 (18)
C10—N1—C1—C2	24.9 (2)	N1—C7—C8—N2	−57.36 (18)
C7—N1—C1—C2	−173.79 (16)	C8—N2—C9—C10	−54.97 (19)
N1—C1—C2—C3	178.48 (17)	C1—N1—C10—C9	103.02 (19)
C6—C1—C2—C3	0.7 (3)	C7—N1—C10—C9	−60.34 (19)
C1—C2—C3—C4	−0.2 (3)	N2—C9—C10—N1	56.90 (19)
C2—C3—C4—C5	−0.7 (3)	O3—C11—C12A—F2A	139.1 (3)
C2—C3—C4—N3	179.71 (17)	O4—C11—C12A—F2A	−45.3 (4)
O1—N3—C4—C3	−4.9 (3)	O3—C11—C12A—F3A	−103.0 (3)
O2—N3—C4—C3	175.97 (18)	O4—C11—C12A—F3A	72.6 (4)
O1—N3—C4—C5	175.49 (18)	O3—C11—C12A—F1A	23.5 (5)
O2—N3—C4—C5	−3.6 (3)	O4—C11—C12A—F1A	−160.9 (3)
C3—C4—C5—C6	1.1 (3)	O3—C11—C12B—F3B	98.1 (11)
N3—C4—C5—C6	−179.33 (15)	O4—C11—C12B—F3B	−69.4 (12)
C4—C5—C6—C1	−0.6 (3)	O3—C11—C12B—F1B	−146.3 (10)
N1—C1—C6—C5	−178.08 (16)	O4—C11—C12B—F1B	46.2 (12)
C2—C1—C6—C5	−0.3 (3)	O3—C11—C12B—F2B	−50.7 (12)
C1—N1—C7—C8	−102.40 (18)	O4—C11—C12B—F2B	141.8 (10)

### 4-(4-Nitrophenyl)piperazin-1-ium trifluoroacetate (I) Hydrogen-bond geometry (Å, º)

**Table:**

*D*—H···*A*	*D*—H	H···*A*	*D*···*A*	*D*—H···*A*
N2—H21···O3	0.90 (2)	1.98 (2)	2.844 (2)	161 (2)
N2—H22···O4^i^	0.87 (2)	1.93 (2)	2.786 (2)	165 (2)
C7—H7*A*···O3^i^	0.97	2.53	3.492 (2)	169

Symmetry code: (i) *x*+1, *y*, *z*.

### 4-(4-Nitrophenyl)piperazin-1-ium trichloroacetate (II) Crystal data

**Table:**

C_10_H_14_N_3_O_2_^+^·C_2_Cl_3_O_2_^−^	*F*(000) = 760
*M**_r_* = 370.61	*D*_x_ = 1.603 Mg m^−^^3^
Monoclinic, *P*2_1_/*c*	Mo *K*α radiation, λ = 0.71073 Å
*a* = 11.7825 (5) Å	Cell parameters from 2813 reflections
*b* = 6.6142 (3) Å	θ = 3.0–27.8°
*c* = 20.3271 (9) Å	µ = 0.62 mm^−^^1^
β = 104.173 (4)°	*T* = 293 K
*V* = 1535.91 (12) Å^3^	Prism, brown
*Z* = 4	0.48 × 0.44 × 0.40 mm

### 4-(4-Nitrophenyl)piperazin-1-ium trichloroacetate (II) Data collection

**Table:**

Oxford Diffraction Xcalibu with Sapphire CCD diffractometer	2068 reflections with *I* > 2σ(*I*)
Radiation source: Enhance (Mo) X-ray Source	*R*_int_ = 0.013
Rotation method data acquisition using ω scans.	θ_max_ = 25.4°, θ_min_ = 3.0°
Absorption correction: multi-scan (CrysAlis RED; Oxford Diffraction (2009)	*h* = −14→13
*T*_min_ = 0.840, *T*_max_ = 1.000	*k* = −7→6
5078 measured reflections	*l* = −24→14
2802 independent reflections	

### 4-(4-Nitrophenyl)piperazin-1-ium trichloroacetate (II) Refinement

**Table:**

Refinement on *F*^2^	Hydrogen site location: mixed
Least-squares matrix: full	H atoms treated by a mixture of independent and constrained refinement
*R*[*F*^2^ > 2σ(*F*^2^)] = 0.036	*w* = 1/[σ^2^(*F*_o_^2^) + (0.0553*P*)^2^ + 0.1937*P*] where *P* = (*F*_o_^2^ + 2*F*_c_^2^)/3
*wR*(*F*^2^) = 0.104	(Δ/σ)_max_ < 0.001
*S* = 1.09	Δρ_max_ = 0.25 e Å^−^^3^
2802 reflections	Δρ_min_ = −0.28 e Å^−^^3^
236 parameters	Extinction correction: *SHELXL2019/2* (Sheldrick, 2015b), Fc^*^=kFc[1+0.001xFc^2^λ^3^/sin(2θ)]^-1/4^
35 restraints	Extinction coefficient: 0.0084 (12)
Primary atom site location: structure-invariant direct methods	

### 4-(4-Nitrophenyl)piperazin-1-ium trichloroacetate (II) Special details

**Table:**

Geometry. All esds (except the esd in the dihedral angle between two l.s. planes) are estimated using the full covariance matrix. The cell esds are taken into account individually in the estimation of esds in distances, angles and torsion angles; correlations between esds in cell parameters are only used when they are defined by crystal symmetry. An approximate (isotropic) treatment of cell esds is used for estimating esds involving l.s. planes.

### 4-(4-Nitrophenyl)piperazin-1-ium trichloroacetate (II) Fractional atomic coordinates and isotropic or equivalent isotropic displacement parameters (Å^2^)

**Table:**

	*x*	*y*	*z*	*U*_iso_*/*U*_eq_	Occ. (<1)
O1	1.25261 (14)	0.5140 (3)	0.20561 (10)	0.0741 (5)	
O2	1.24364 (14)	0.5039 (2)	0.30936 (10)	0.0669 (5)	
N1	0.70718 (14)	0.5089 (3)	0.16441 (8)	0.0449 (4)	
N2	0.47368 (15)	0.3654 (3)	0.13414 (9)	0.0428 (4)	
N3	1.19635 (15)	0.5092 (2)	0.24883 (10)	0.0477 (5)	
C1	0.82694 (16)	0.5103 (2)	0.18508 (9)	0.0332 (4)	
C2	0.89695 (17)	0.5088 (3)	0.13812 (10)	0.0410 (5)	
H2	0.861345	0.508580	0.091922	0.049*	
C3	1.01632 (18)	0.5077 (3)	0.15913 (11)	0.0432 (5)	
H3	1.061159	0.506564	0.127313	0.052*	
C4	1.07033 (16)	0.5084 (3)	0.22736 (10)	0.0369 (4)	
C5	1.00483 (17)	0.5083 (3)	0.27476 (10)	0.0379 (5)	
H5	1.041717	0.507704	0.320778	0.046*	
C6	0.88472 (17)	0.5091 (3)	0.25404 (9)	0.0370 (4)	
H6	0.840985	0.508872	0.286420	0.044*	
C7	0.63004 (17)	0.5611 (3)	0.20782 (11)	0.0452 (5)	
H7A	0.590860	0.688009	0.192889	0.054*	
H7B	0.675741	0.578246	0.254138	0.054*	
C8	0.54056 (16)	0.3977 (3)	0.20527 (10)	0.0425 (5)	
H8A	0.579293	0.273110	0.223440	0.051*	
H8B	0.487520	0.436125	0.232840	0.051*	
C9	0.55236 (18)	0.3230 (3)	0.08906 (10)	0.0466 (5)	
H9A	0.506515	0.312758	0.042528	0.056*	
H9B	0.591456	0.194556	0.101561	0.056*	
C10	0.64214 (18)	0.4869 (3)	0.09421 (10)	0.0489 (5)	
H10A	0.695324	0.453110	0.066221	0.059*	
H10B	0.603791	0.613458	0.077935	0.059*	
O3	0.39831 (14)	−0.0360 (2)	0.13071 (8)	0.0613 (5)	
O4	0.35547 (15)	−0.3109 (2)	0.06595 (8)	0.0619 (5)	
C11	0.33900 (17)	−0.1382 (3)	0.08446 (10)	0.0412 (5)	
C12	0.22438 (17)	−0.0367 (3)	0.04153 (9)	0.0394 (5)	
Cl1A	0.1882 (4)	0.1717 (9)	0.0837 (2)	0.0708 (9)	0.494 (15)
Cl2A	0.2502 (7)	0.0289 (11)	−0.0355 (3)	0.0911 (17)	0.494 (15)
Cl3A	0.1076 (4)	−0.2089 (7)	0.0240 (3)	0.0746 (11)	0.494 (15)
Cl1B	0.2023 (4)	0.2189 (9)	0.0640 (4)	0.0790 (16)	0.506 (15)
Cl2B	0.2284 (6)	−0.0136 (10)	−0.0438 (2)	0.0839 (13)	0.506 (15)
Cl3B	0.1049 (4)	−0.1756 (13)	0.0517 (5)	0.100 (2)	0.506 (15)
H21	0.4277 (18)	0.263 (3)	0.1340 (11)	0.047 (6)*	
H22	0.4300 (19)	0.468 (3)	0.1176 (11)	0.061 (7)*	

### 4-(4-Nitrophenyl)piperazin-1-ium trichloroacetate (II) Atomic displacement parameters (Å^2^)

**Table:**

	*U*^11^	*U*^22^	*U*^33^	*U*^12^	*U*^13^	*U*^23^
O1	0.0405 (9)	0.0979 (15)	0.0875 (13)	0.0003 (9)	0.0227 (9)	−0.0052 (10)
O2	0.0443 (9)	0.0759 (12)	0.0697 (11)	−0.0015 (8)	−0.0068 (8)	−0.0106 (9)
N1	0.0330 (9)	0.0664 (12)	0.0364 (9)	−0.0058 (8)	0.0106 (7)	−0.0123 (8)
N2	0.0334 (9)	0.0375 (10)	0.0536 (11)	−0.0019 (8)	0.0033 (8)	0.0032 (8)
N3	0.0357 (9)	0.0363 (10)	0.0689 (13)	0.0012 (7)	0.0087 (9)	−0.0069 (8)
C1	0.0344 (10)	0.0273 (9)	0.0388 (10)	−0.0020 (8)	0.0106 (8)	−0.0025 (8)
C2	0.0406 (11)	0.0483 (12)	0.0350 (10)	0.0030 (9)	0.0108 (8)	0.0016 (9)
C3	0.0405 (11)	0.0439 (12)	0.0491 (12)	0.0042 (9)	0.0181 (9)	0.0022 (9)
C4	0.0333 (10)	0.0252 (9)	0.0506 (12)	−0.0004 (8)	0.0076 (8)	−0.0017 (8)
C5	0.0429 (11)	0.0286 (10)	0.0395 (10)	−0.0014 (8)	0.0046 (9)	−0.0032 (8)
C6	0.0423 (11)	0.0349 (10)	0.0359 (10)	−0.0026 (8)	0.0136 (8)	−0.0024 (8)
C7	0.0348 (10)	0.0567 (12)	0.0464 (12)	−0.0050 (9)	0.0144 (9)	−0.0130 (10)
C8	0.0368 (10)	0.0466 (12)	0.0442 (11)	0.0020 (9)	0.0104 (8)	0.0017 (9)
C9	0.0449 (12)	0.0509 (12)	0.0388 (11)	0.0026 (10)	0.0001 (9)	−0.0060 (9)
C10	0.0391 (11)	0.0675 (15)	0.0386 (11)	−0.0004 (10)	0.0066 (8)	−0.0006 (10)
O3	0.0579 (10)	0.0507 (9)	0.0598 (10)	−0.0039 (8)	−0.0152 (8)	0.0023 (7)
O4	0.0640 (10)	0.0505 (9)	0.0616 (10)	0.0210 (8)	−0.0030 (8)	−0.0044 (8)
C11	0.0383 (11)	0.0456 (12)	0.0375 (11)	0.0031 (9)	0.0054 (8)	0.0057 (9)
C12	0.0386 (11)	0.0401 (10)	0.0378 (11)	0.0010 (9)	0.0062 (8)	−0.0024 (8)
Cl1A	0.0801 (15)	0.0735 (19)	0.0577 (13)	0.0376 (13)	0.0150 (12)	−0.0120 (11)
Cl2A	0.121 (3)	0.099 (3)	0.067 (3)	0.040 (2)	0.050 (2)	0.053 (2)
Cl3A	0.0501 (11)	0.0651 (12)	0.095 (2)	−0.0146 (8)	−0.0082 (13)	0.0026 (15)
Cl1B	0.0603 (14)	0.0597 (17)	0.095 (3)	0.0272 (12)	−0.0224 (15)	−0.0358 (18)
Cl2B	0.100 (2)	0.107 (3)	0.0388 (11)	0.060 (2)	0.0071 (13)	0.0062 (15)
Cl3B	0.0427 (9)	0.115 (3)	0.137 (4)	−0.0141 (15)	0.0102 (19)	0.063 (3)

### 4-(4-Nitrophenyl)piperazin-1-ium trichloroacetate (II) Geometric parameters (Å, º)

**Table:**

O1—N3	1.224 (3)	C7—C8	1.502 (3)
O2—N3	1.221 (2)	C7—H7A	0.9700
N1—C1	1.370 (2)	C7—H7B	0.9700
N1—C10	1.452 (2)	C8—H8A	0.9700
N1—C7	1.455 (2)	C8—H8B	0.9700
N2—C9	1.481 (3)	C9—C10	1.501 (3)
N2—C8	1.483 (2)	C9—H9A	0.9700
N2—H21	0.867 (16)	C9—H9B	0.9700
N2—H22	0.870 (16)	C10—H10A	0.9700
N3—C4	1.442 (2)	C10—H10B	0.9700
C1—C6	1.400 (3)	O3—C11	1.228 (2)
C1—C2	1.406 (3)	O4—C11	1.233 (2)
C2—C3	1.366 (3)	C11—C12	1.568 (3)
C2—H2	0.9300	C12—Cl2A	1.722 (4)
C3—C4	1.377 (3)	C12—Cl1A	1.730 (5)
C3—H3	0.9300	C12—Cl3B	1.736 (4)
C4—C5	1.374 (3)	C12—Cl2B	1.753 (5)
C5—C6	1.374 (3)	C12—Cl3A	1.754 (5)
C5—H5	0.9300	C12—Cl1B	1.787 (4)
C6—H6	0.9300		
			
C1—N1—C10	123.91 (16)	H7A—C7—H7B	108.1
C1—N1—C7	124.18 (17)	N2—C8—C7	109.76 (16)
C10—N1—C7	111.23 (17)	N2—C8—H8A	109.7
C9—N2—C8	111.52 (15)	C7—C8—H8A	109.7
C9—N2—H21	109.6 (14)	N2—C8—H8B	109.7
C8—N2—H21	107.4 (14)	C7—C8—H8B	109.7
C9—N2—H22	108.0 (16)	H8A—C8—H8B	108.2
C8—N2—H22	112.8 (16)	N2—C9—C10	110.93 (16)
H21—N2—H22	107 (2)	N2—C9—H9A	109.5
O2—N3—O1	122.05 (19)	C10—C9—H9A	109.5
O2—N3—C4	119.16 (19)	N2—C9—H9B	109.5
O1—N3—C4	118.79 (19)	C10—C9—H9B	109.5
N1—C1—C6	121.24 (17)	H9A—C9—H9B	108.0
N1—C1—C2	121.54 (17)	N1—C10—C9	109.53 (17)
C6—C1—C2	117.21 (18)	N1—C10—H10A	109.8
C3—C2—C1	121.20 (18)	C9—C10—H10A	109.8
C3—C2—H2	119.4	N1—C10—H10B	109.8
C1—C2—H2	119.4	C9—C10—H10B	109.8
C2—C3—C4	120.09 (19)	H10A—C10—H10B	108.2
C2—C3—H3	120.0	O3—C11—O4	129.89 (19)
C4—C3—H3	120.0	O3—C11—C12	116.20 (18)
C5—C4—C3	120.39 (18)	O4—C11—C12	113.91 (17)
C5—C4—N3	120.10 (18)	C11—C12—Cl2A	107.1 (3)
C3—C4—N3	119.52 (18)	C11—C12—Cl1A	110.38 (19)
C4—C5—C6	119.88 (18)	Cl2A—C12—Cl1A	111.7 (2)
C4—C5—H5	120.1	C11—C12—Cl3B	108.7 (2)
C6—C5—H5	120.1	C11—C12—Cl2B	111.1 (2)
C5—C6—C1	121.24 (18)	Cl3B—C12—Cl2B	112.5 (3)
C5—C6—H6	119.4	C11—C12—Cl3A	111.2 (2)
C1—C6—H6	119.4	Cl2A—C12—Cl3A	106.4 (3)
N1—C7—C8	110.22 (17)	Cl1A—C12—Cl3A	110.0 (2)
N1—C7—H7A	109.6	C11—C12—Cl1B	114.97 (18)
C8—C7—H7A	109.6	Cl3B—C12—Cl1B	107.3 (2)
N1—C7—H7B	109.6	Cl2B—C12—Cl1B	102.2 (3)
C8—C7—H7B	109.6		
			
C10—N1—C1—C6	−172.70 (18)	C10—N1—C7—C8	60.7 (2)
C7—N1—C1—C6	17.6 (3)	C9—N2—C8—C7	54.7 (2)
C10—N1—C1—C2	6.0 (3)	N1—C7—C8—N2	−57.0 (2)
C7—N1—C1—C2	−163.77 (18)	C8—N2—C9—C10	−54.9 (2)
N1—C1—C2—C3	−179.19 (18)	C1—N1—C10—C9	129.3 (2)
C6—C1—C2—C3	−0.5 (3)	C7—N1—C10—C9	−59.8 (2)
C1—C2—C3—C4	−0.1 (3)	N2—C9—C10—N1	56.4 (2)
C2—C3—C4—C5	0.6 (3)	O3—C11—C12—Cl2A	105.8 (4)
C2—C3—C4—N3	−179.41 (18)	O4—C11—C12—Cl2A	−74.1 (4)
O2—N3—C4—C5	2.1 (3)	O3—C11—C12—Cl1A	−16.0 (3)
O1—N3—C4—C5	−178.32 (17)	O4—C11—C12—Cl1A	164.1 (3)
O2—N3—C4—C3	−177.92 (18)	O3—C11—C12—Cl3B	−117.0 (5)
O1—N3—C4—C3	1.7 (3)	O4—C11—C12—Cl3B	63.2 (5)
C3—C4—C5—C6	−0.5 (3)	O3—C11—C12—Cl2B	118.7 (3)
N3—C4—C5—C6	179.50 (16)	O4—C11—C12—Cl2B	−61.1 (3)
C4—C5—C6—C1	−0.1 (3)	O3—C11—C12—Cl3A	−138.3 (3)
N1—C1—C6—C5	179.29 (17)	O4—C11—C12—Cl3A	41.8 (3)
C2—C1—C6—C5	0.6 (3)	O3—C11—C12—Cl1B	3.3 (5)
C1—N1—C7—C8	−128.4 (2)	O4—C11—C12—Cl1B	−176.6 (4)

### 4-(4-Nitrophenyl)piperazin-1-ium trichloroacetate (II) Hydrogen-bond geometry (Å, º)

**Table:**

*D*—H···*A*	*D*—H	H···*A*	*D*···*A*	*D*—H···*A*
N2—H21···O3	0.87 (2)	2.01 (2)	2.795 (2)	151 (2)
N2—H21···Cl1*A*	0.87 (2)	2.82 (2)	3.510 (5)	138 (2)
N2—H22···O4^i^	0.87 (2)	1.89 (2)	2.738 (2)	167 (2)

Symmetry code: (i) *x*, *y*+1, *z*.
